# IMM0306, a fusion protein of CD20 mAb with the CD47 binding domain of SIRPα, exerts excellent cancer killing efficacy by activating both macrophages and NK cells via blockade of CD47-SIRPα interaction and FcɣR engagement by simultaneously binding to CD47 and CD20 of B cells

**DOI:** 10.1038/s41375-022-01805-9

**Published:** 2022-12-27

**Authors:** Jifeng Yu, Song Li, Dianze Chen, Dandan Liu, Huiqin Guo, Chunmei Yang, Wei Zhang, Li Zhang, Gui Zhao, Xiaoping Tu, Liang Peng, Sijin Liu, Xing Bai, Yongping Song, Zhongxing Jiang, Ruliang Zhang, Wenzhi Tian

**Affiliations:** 1 Department of Hematology, the First Affiliated Hospital of Zhengzhou University, Zhengzhou, 450052 Henan China; 2 ImmuneOnco Biopharmaceuticals (Shanghai) Co., Ltd., Shanghai, 201203 China

**Keywords:** Immunotherapy, Drug development, Preclinical research

## To the Editor:

CD47, as the “don’t eat me” signal, interacts with the inhibitory signal-regulatory protein alpha (SIRPα) receptor expressed by myeloid cells and activated NK cells [1] and is overexpressed in different malignancies, causing tumors to escape the phagocytosis of macrophages [2]. CD47 was identified as the main macrophage checkpoint, and blocking CD47 can make macrophages phagocytize tumor cells and produce therapeutic clearance. Recent progress has been made in targeting CD47 for cancer immunotherapy [1], and several CD47/SIRPα axis inhibitors have been developed for clinical trials with a favorable antitumor response in solid tumors and hematological malignancies [1, 3, 4]. However, the clinical development of Fc-active anti-CD47 antibodies is hindered by the potential adverse events of hematological dose-limiting toxicity due to the broad expression of CD47 on blood cells, including erythrocytes and platelets [5, 6]. The improved priming administration scheme of anti-CD47 molecules in combination with other agents promoting the expression of the prophagocytic signal on tumor cells has been used to avoid this adverse effect and demonstrated effective results [3, 7, 8].

Different bispecific antibodies (BsAb) or fusion proteins targeting both CD47 and B cell antigens, such as CD20 and CD19, were generated to target and deplete B cells *via* multiple antibody-mediated mechanisms [5, 9]. CD20-CD47SL, a CD47xCD20 BsAb, was observed to selectively bind to dual antigen-expressing lymphoma cells in the presence of an “antigen sink” of RBCs and recapitulated the synergistic effects of anti-CD47 antibody and rituximab combinations in mouse models of NHL [10]. Another anti-CD47/CD20 BsAb showed potent antagonism of the CD47/SIRPα pathway [11]. RTX-CD47, a CD20-targeting scFv antibody fragment derived from rituximab fused in tandem with a CD47-blocking scFv, demonstrated therapeutic anticancer activity in the mouse model of B-cell tumors [12]. Meanwhile, a fully human BsAb targeting CD19 and CD47, NI-1701, demonstrated therapeutic effect in patients with B cell malignancies refractory/resistant to anti-CD20 targeted therapy [5]. Another CD47xCD19 BsAb triggers recruitment and activation of innate immune effector cells in a B-cell lymphoma xenograft model [13]. The role of CD47xCD19 co-ligation in inhibiting B cell proliferation illuminates a novel approach that may provide a therapeutic benefit in settings of autoimmunity and B cell malignancies. Here, we describe the anti-tumor mechanism of IMM0306, a fusion protein of CD20 monoclonal antibody (mAb) with the CD47 binding domain of SIRPα, by activating both macrophages and NK cells via blockade of CD47-SIRPα interaction and FcɣR engagement by simultaneously binding to CD47 and CD20 of B cells in different mouse xenograft tumor models.

**Fig. 1:**
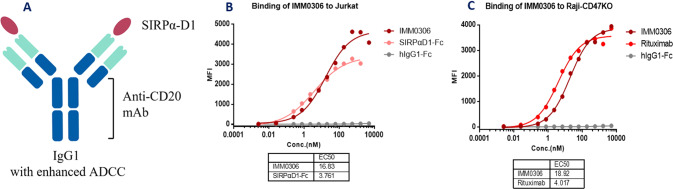
The molecule structure and binding activities of IMM0306. **A** A representation of the structure of IMM0306, a fusion protein of CD20 mAb with the CD47 binding domain of SIRPα (V2) extracellular segment domain 1 (D1) on both heavy chains. It simultaneously binds to CD47 and CD20 of B cells. **B** IMM0306 binding activity on Jurkat cells: Cultured Jurkat cells were stained with IMM0306, SIRPα-Fc and hIgG1-Fc, followed by staining with a secondary antibody anti-human IgG (Fc)–FITC. Then the cells were analyzed by flow cytometry to measure the binding ability on Jurkat cells. The data was further analyzed with GraphPad Prism 8.0 software. The results showed that IMM0306 has strong binding activity on Jurkat cells. **C** IMM0306 binding activity on Raji-CD47KO cells: The Raji-CD47KO cell line, modified from the Raji cell line with human CD47 knockout, was established in-house with the standard gene knockout procedures. Cultured Raji-CD47KO cells were stained with IMM0306, rituximab, and hIgG1-Fc, followed by staining with the secondary antibody anti-human IgG (Fc)–FITC. Then the cells were analyzed by flow cytometry to measure the binding ability of Raji-CD47KO cells. The data was further analyzed with GraphPad Prism 8.0 software. The results showed that IMM0306 has strong binding activity on Raji-CD47KO cells.

IMM0306 is a fusion protein of CD20 mAb with the CD47 binding domain of SIRPα on both heavy chains (Fig. 1A). IMM0306 was constructed and produced using an in-house developed CHO-K1 cell expression system. The binding activity of IMM0306 was analyzed by flow cytometry. The phagocytosis and in vitro anti-tumor activity of IMM0306 were evaluated by antibody-dependent cellular phagocytosis (ADCP), antibody-dependent cell-mediated cytotoxicity (ADCC), and complement-dependent cytotoxicity (CDC) assays. In vivo mouse tumor model studies were used to explore therapeutic efficacy as well as the mechanism of action.

In vitro binding assays revealed that IMM0306 has a high affinity for the CD47 target on Jurkat cells, the CD20 target on Raji-CD47KO cells, and the simultaneous binding of CD47/CD20 double targets (Figs. 1B, C, S1, S2, and S3), with EC50 values of 16.83 nM, 18.92 nM, and 11.48 nM, respectively. A dual binding assay revealed that IMM0306 can simultaneously bind CD20-positive cells (Raji-CD47KO) and CD47-positive cells (Jurkat) with an EC50 of 0.02462ug/ml. The Biacore assay showed that the affinity of IMM0306 for CD20 and CD47 targets was 2.45 nM and 4.91 nM, respectively (Fig. S4). By blocking the SIRPa/CD47 pathway, IMM0306 significantly inhibited the apoptosis of Jurkat-CSR cells, with an IC50 of 4.046 nM (Fig. S7C). Jurkat-CSR is an in-house developed cell line from the Jurkat cell line with CSR including the human extracellular SIRPα domain sequentially connected by CD8a-hinge, CD28-TMD/ICD, and CD3ζ signal domains, and human CD47 knockout established with the standard gene overexpression and gene knockout procedures. IMM0306 binds to various cells, including Raji, Daudi, Jeko-1, Ramos, SU-DHL-4, SU-DHL-10, human peripheral mononuclear cells (PBMC), and monkey PBMC (Figs. S5, S6). IMM0306 barely binds to human red blood cells (RBC), while conventional CD47 mAb hB6H12 binds to RBC with high affinity. This minimal binding to RBC has been further confirmed in blood samples from 100 donors (Fig. S7A). Conventional CD47 mAb hB6H12 induces hemagglutination at an optimal concentration. IMM0306, together with rituximab and the negative control, does not show such activity (Fig. S8). In a cross-reactivity assay by enzyme-linked immunity assay, IMM0306 binds to human and cynomolgus CD47 but not mouse or rat CD47 (Fig. S7B). Collectively, as a CD47xCD20 mAb trap, IMM0306 will not cause serious anemia side effects because the receptor segment bound to CD47 does not bind to red blood cells.

The ADCC assay showed that IMM0306 has strong ADCC induced by activating NK cells through enhanced Fc/FcɣRIIIa interaction (Fig. S9A). Strong ADCP is induced by fully activating macrophages through simultaneously blocking the “don’t eat me” signal of CD47-SIRPα interaction and enhancing Fc-FcɣRIIa interaction (Figs. S9B, S10), and strong CDC is induced as a result of complement cascade activation (Fig. S9C). IMM0306 can induce stronger ADCC and lower CDC activities than rituximab on various tumor cells, including Raji, Daudi, Jeko-1, Ramos, SU-DHL-4, and SU-DHL-10 (Figs. S11, S12), but lower activities against normal cells, whereas the CD20 arm is the major contributor of IMM0306’s ADCC and CDC, suggesting less on-target off-tumor toxicity. Furthermore, IMM0306 can strongly induce macrophages from different donors to phagocytose Raji, Jeko-1, and Ramos cells. IMM0306 is more potent than rituximab monotherapy at a much lower dose level and the combination therapy of IMM01 and rituximab at a comparable dose level. Collectively, the recombinant protein has much higher affinity for CD20 than that of CD47, so it will preferentially combine with CD20 positive lymphoma cells, so that the CD47 binding fragment will be pulled to the same CD20xCD47 double positive cells, thus avoiding the binding to the CD47 positive cells in the blood, reducing the toxicity related to CD47 targets.

**Fig. 2:**
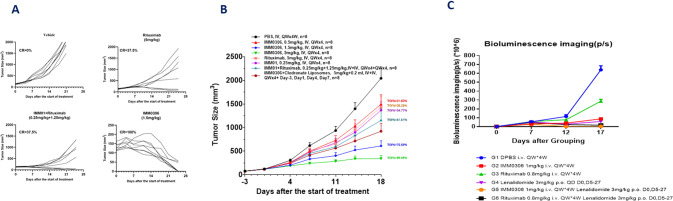
In vivo efficacy of IMM0306. **A** The cell inoculation method was used to create human leukemia cell Daudi CB17-SCID mice xenograft subcutaneous tumor models: tumor cells were collected in logarithmic growth stages and subcutaneously inoculated into SCID mice with 2 × 106/0.1 ml/mouse cell suspension in 1xPBS with Matrigel at a 1:1 ratio at a cell concentration of 2 × 10^7^/ml. When the tumor volumes reached 100–200 mm^3^, the animals were randomly divided into different groups with the tumor difference between each group being less than 10% of the mean value. Different agents were administered to the different groups of mice. The tumor volumes and the body weight of the animals were measured three times a week, and the clinical symptoms were observed and recorded once a day. The results revealed that IMM0306 exerts excellent cancer killing efficacy by activating both macrophages and NK cells *via* blockade of CD47-SIRPα interaction and FcɣR engagement. After administration of IMM0306 at 1.5 mg/kg for 3 weeks, 100% of the SCID mice in the Daudi xenograft model achieved complete remission (CR). **B** CB17-SCID mice xenograft orthotropic Raji tumor model was established by inoculating 0.2 ml cell suspension at the concentration of 5 × 10^6^/mouse by caudal vein. Three days after inoculation, animals were randomly divided into different groups with the body weight difference between each group being less than 10% of the mean value. Starting on day 0, the drugs were administered according to the weight of the animals. During the administration, if the weight of individual animals decreases by more than 15% compared to day 0 (BWL ≧15%), the drug will be stopped until the weight of animals recovers (BWL ≦ 15%). During the experiment, tumor volumes and the body weight of the animals were measured three times a week, and the clinical symptoms were observed and recorded once a day, and the TGI values were calculated. The results showed that IMM0306 is more potent than rituximab and the combo of two single agents in tumor-inhibition, with a TGI of 92.7% with IMM0306 versus 27.6% with rituximab, respectively. **C** Raji-luc inoculated CB17-SCID mice in situ to evaluate the therapeutic effect of IMM0306, rituximab, lenalidomide, and their combinations. CB17 SCID mice were inoculated with Raji-luc human lymphoma cells via tail vein injection. On the 14th day after inoculation, the mice were intraperitoneally injected with the fluorescent substrate D-Luciferin. About 15 min later, the mice were anesthetized with 3–4% isoflurane, and the fluorescence intensity of the mice was detected with a live imaging system (PerkinElmer, Lumina XRMS). During the period, the weight of the mice was monitored, and the clinical symptoms were recorded. The mice were imaged in vivo on days 7, 12 and 17 after grouping to detect the tumor fluorescence intensity. The data on the 12th day of in vivo imaging showed that the tumor fluorescence intensity of mice in the solvent group (non-drug treatment group) was significantly increased after tumor cells were inoculated, and the tumor fluorescence intensity of mice in the drug treatment group was weaker than that in the solvent group, showing a certain therapeutic effect. The tumor fluorescence intensity of IMM0306 combined with lenalidomide was the lowest, which was significantly better than that of any single drug group of IMM0306 and lenalidomide, which was consistent with the weight and clinical symptom data of mice. The data showed that the three drugs all showed a certain degree of anti-tumor effect, but the therapeutic effect of IMM0306 combined with lenalidomide was significantly better than that of any single drug, and better than that of rituximab combined with lenalidomide.

Furthermore, in vivo mouse model results revealed that IMM0306 exerts excellent cancer killing efficacy by activating both macrophages and NK cells *via* blockade of CD47-SIRPα interaction and FcɣR engagement. After administration of IMM0306 at 1.5 mg/kg for 3 weeks, 100% of the SCID mice in the Daudi xenograft model achieved complete remission (CR) (Figs. 2A, S13), and tumors could be eliminated. In the Raji xenograft model in SCID mice, IMM0306 is more potent than rituximab and the combo of two single agents in tumor-inhibition, with a tumor growth inhibition (TGI) of 89.98% with IMM0306 versus 26.26% with rituximab, respectively, after administration of IMM0306 5 mg/kg for 4 weeks (Fig. 2B). These collective results demonstrated that IMM0306 has strong anti-tumor efficacy against blood tumors such as Daudi and Raji, suggesting the therapeutic potential of IMM0306 in the treatment of patients with hematological malignancies. In addition, the in vivo efficacy of IMM0306 combined with lenalidomide in a lymphoma orthotopic model demonstrated the therapeutic effect of IMM0306 combined with lenalidomide was significantly better than that of any single drug or rituximab combined with lenalidomide (Figs. 2C, S14).

In conclusion, our data demonstrated that IMM0306, a fusion protein of CD20 mAb with the CD47 binding domain of SIRPα, exerts excellent cancer killing efficacy by activating both macrophages and NK cells via blockade of CD47-SIRPα interaction and FcɣR engagement by simultaneously binding to CD47 and CD20 of B cells. IMM0306 has stronger ADCC activity compared to rituximab, stronger ADCC and ADCP compared to SIRPα-Fc, very low binding activities on human RBCs to avoid potential human cell toxicities, and a higher affinity to CD20 for strong tumor cell killing. IMM0306 can be used as monotherapy or in combination with other targeted immune checkpoint inhibitors. Combining with our previous study [14], we believed that for therapeutic purposes by targeting the CD47/SIRPα signal pathway, two prerequisites must be met for full activation of macrophages, blocking of the CD47 “don’t eat me” signal and activation of the Fc-receptor “eat me” signal. Either one of the two prerequisites can only exhibit limited macrophage activation. We conclude the mechanism of action of IMM0306 by blocking the CD47 “don’t eat me” signal and activating the Fc-receptor “eat me” signal of CD20 with a strong ADCP to make the B-cell fully killed, whereas CD47 mAbs and rituximab can only either block the CD47 “don’t eat me” signal or activate the Fc-receptor “eat me” signal of CD20 with limited ADCP to make the limited B-cell killed (Fig. S15). The Fc segment of the recombinant protein in IMM0306 is IgG1 and has been modified by ADCC-enhanced genetic engineering. Therefore, it has strong in vivo efficacy (through ADCC and ADCP). For other CD47 BsAbs, because the CD47 antibody itself combines with red blood cells [1, 15], IgG1 cannot be selected; otherwise, it will have severe anemia toxicity. Therefore, our data revealed that IMM0306 has a safer profile and superior therapeutic potential than other CD47 mAbs and BsAbs.

## Supplementary information

Supplement file and figures S1-S15

## Data Availability

The datasets generated during and/or analysed during the current study are available from the corresponding author on reasonable request.
