# Supplementary material for: IMM0306, a fusion protein of CD20 mAb with the CD47 binding domain of SIRPα, exerts excellent cancer killing efficacy by activating both macrophages and NK cells via blockade of CD47-SIRPα interaction and FcɣR engagement by simultaneously binding to CD47 and CD20 of B cells
Source: Leukemia. 2022 Dec 27;37(3):695–8. doi: 10.1038/s41375-022-01805-9 (PMC9991911; doi:10.1038/s41375-022-01805-9)

**Supplement file and figures S1-S15**

**Figure S1.** **IMM0306 can simultaneously bind to CD20 and CD47 targets: Raji CD47KO + IMM0306 with different concentrations of CD47-Fc****.**

Testing results with fixed IMM0306 concentration with different CD47-Fc concentrations: Raji CD47KO+IMM0306+CD47-Fc at different concentrations were used to detect the binding activity. 100ul of Raji-CD47KO cells at 0.5x10^6^/ml and 100ul of IMM0306 at concentration of 300nM were mixed and incubated at 4℃ for 45 min. After washing once (3000rpm, 3min) with 1% BSA-PBS 1ml, 100ul of different concentrations of biotin conjugated CD47-FC was added and incubated at 4℃ for 45 min. After washing once with 1% BSA-PBS 1ml, 100ul of FITC conjugated Streptavidin was added and incubated at 4℃ for 45 min. After washing once with 1% BSA-PBS 1ml, samples were tested by flow cytometry.

**Figure S2.** **IMM0306 can simultaneously bind to CD20 and CD47 targets: Raji CD47KO +CD47-Fc with different concentrations of IMM0306.**

Testing results with fixed CD47-Fc concentrations with different IMM0306 concentrations: Raji CD47KO+IMM0306+CD47-Fc at different concentrations were used to detect the binding activity. 100ul of Raji-CD47KO cells at 0.5x10^6^/ml and 100ul of IMM0306 at different concentrations were mixed and incubated at 4℃ for 45 min. After washing once (3000rpm, 3min) with 1% BSA-PBS 1ml, 100ul of 100nM biotin conjugated CD47-FC were added and incubated at 4℃ for 45 min. After washing once, 100ul of FITC-conjugated streptavidin was added and incubated at 4℃ for 45 min. After washing, samples were tested by flow cytometry.

**Figure S3.** **Dual binding of IMM0306 on Raji-CD47KO and Jurkat cells. IMM0306 can simultaneously bind CD20-positive cells (Raji-CD47KO) and CD47-positive cells (Jurkat).**

Raji CD47KO cells (CD20+/CD47 -) and Jurkat cells (CD20 -/CD47+) were adjusted to the density of 2x10^5^/ml. 50ul Raji-CD47KO cells, 50ul Jurkat cells, and 50ul IMM0306 antibodies were mixed and incubated in 37℃ for 2h. After incubation, the cell suspension was gently mixed, and the cell clustering rate was analyzed with the cell counter (CountStar IC1000, Thermo Fisher Scientific).

**Figure S4. Biacore assay: The affinity of IMM0306 for CD20 and CD47 targets was 2.45E-09 and 4.908E-09, respectively.**


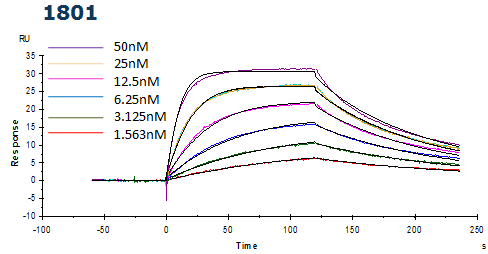

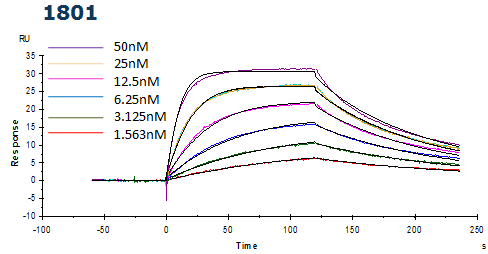

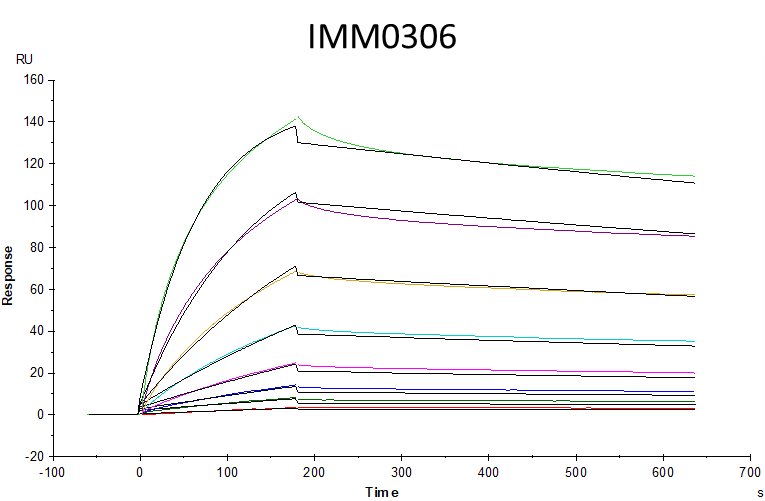

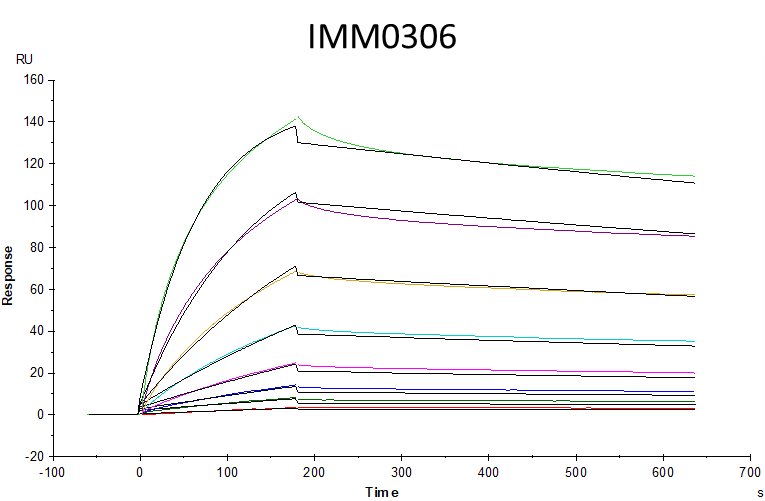


**CD20 Target**

**CD47 Target**

| **Target** | **ka (1/Ms)** | **kd (1/s)** | **KD (M)** |
| --- | --- | --- | --- |
| CD20 | 1.44E+05 | 3.54E-04 | 2.45E-09 |
| CD47 | 2.637E+06 | 1.294E-02 | 4.908E-09 |

**Figure S5. IMM0306 binding to different tumor cells, CD47^+^/CD20^+^ Tumor cells (Raji、Daudi、Jeko-1、Ramos、SU-DHL-4、SU-DHL-10).**

**Figure S6. IMM0306 binding to human and monkey PBMC and different subsets of cells.**

**Human**


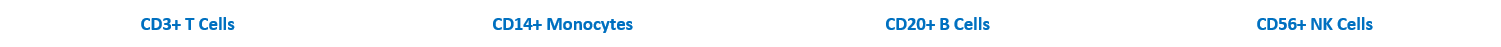


**Monkey**


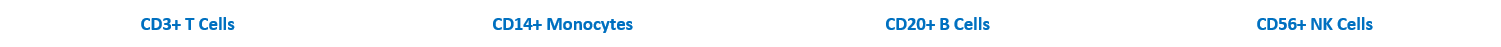


**S7A**

**S7C**

**S7B**

**Figure S7A.** Human red blood cell (RBCs) binding assay: Human RBCs were diluted and incubated with hIgG1-Fc, hB6H12, IMM0306 and rituximab, followed by staining with secondary antibody anti-human IgG (Fc)-FITC (Sigma, cat# F9512). Cells were analyzed by flow cytometry to measure the binding activity of IMM0306. A total of 100 normal donors (62 males and 38 females) with different blood types were tested by flow cytometry. The results showed that IMM0306 has very limited binding activity on human RBC.

**Figure S7B.** Cross-reaction of IMM0306 to different species’ CD47: The standard sandwich ELISA assays were used to measure the cross-reaction with different species’ CD47 by coating IMM0306 on the plates and coating the CD47 on the plates, respectively, followed by the standard detection secondary antibodies. The absorbance values were read at 450 nM. The results were analyzed by GraphPad Prism 8.0 software with four parameters. The results showed that IMM0306 can bind to CD47 in humans and cynomolgus but not in mouse or rat.

**Figure S7C.** SIRPα/CD47 blocking: Jurkat-CSR cells were incubated for 45 minutes with CD47 Fc protein and IMM0306 protein, then co-cultured with the CD47-FC/CD47 antibody mixture for 20 hours. The CCK-8 method was used to detect live Jurkat-CSR cells. The absorbance at 450 nm was measured with the microplate reader. Experimental data was calculated with the following formula:

The results showed that IMM0306 can significantly inhibit the apoptosis induced by CD47 antibodies with an IC50 of 4.046 nM.

**Figure S8 Hemagglutination assay: Conventional CD47 mAb hB6H12 induced hemagglutination at an optimal concentration; IMM0306, together with rituximab and a negative control, did not show such activity.**


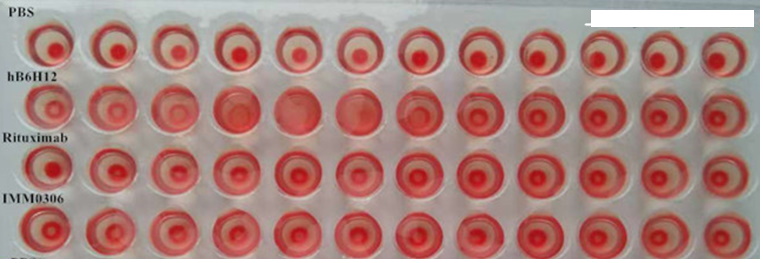

**S9A**

**S9B**

**S9C**

**Figure S9A:** ADCC assay: ADCC assay: Raji-CD20KO and Raji-CD47KO cell lines were established in-house using standard gene knockout procedures from the Raji cell line with human CD20 and CD47 knockout. Raji, Raji-CD20KO and Raji-CD47KO cells were labeled with carboxyfluorescein succinimidyl ester (CSFE) and incubated for 4 hours at 37 °C with 5% CO2 with FcgRIIIA (158V) target-activated NK (FcR-TANKTM) cells (developed in-house). The cells were stained with Propidium Iodide (PI) solution before the flow cytometry method was used to collect the cells and the PI positive staining cells were calculated. ADCC intensity was calculated as follows: Lysis% = (sample% PI positive cell - no antibody% PI positive cell) / (100 - no antibody% PI positive cell) x 100%. The results showed that CD20 knockout can significantly reduce the ADCC effects, suggesting that the CD20 arm is the major contributor to IMM0306’s ADCC. No major ADCC would be expected on normal cells expressing CD47, suggesting less on-target off-tumor toxicity of IMM0306.

**Figure S9B.** ADCP assay: Monocyte isolation and macrophage differentiation were performed using the standard method as described in the supplementary file. The phagocytosis ratio was calculated with the formula: phagocytosis (%) = experimental group phagocytosis (%)– blank control group phagocytosis (%). The results showed that IMM0306 and rituximab can induce strong ADCP by inducing macrophages to phagocytose Raji cells.

**Figure S9C.** CDC assay: Raji, Raji-CD20KO, Raji-CD47KO cells were incubated with different concentrations of IMM0306, and standard rabbit complex complement at 37 ℃ with 5% CO2 for 4 hours, followed by staining with PI solution. The flow cytometry method was used to collect the cells and the PI positive cells were calculated. The calculation of CDC intensity was done by using the following formula: Lysis %= Experimental Sample Lysis %- No Antibody Lysis %. The results showed that CD47 knockout only reduced a limited CDC effect. However, CD20 knockout can significantly reduce the CDC effect, suggesting that the CD20 arm is the major contributor of IMM0306’s CDC effects. Therefore, lower CDC activity would be expected on normal cells expressing CD47, suggesting less on-target off-tumor toxicity of IMM0306.

**Figure S10. IMM0306 can induce macrophages from different donors to phagocytose Raji cells.**

**Figure S11. IMM0306 can induce stronger ADCC activities than rituximab on various tumor cells, including Raji, Daudi, Jeko-1, Ramos, SU-DHL-4, and SU-DHL-10.**


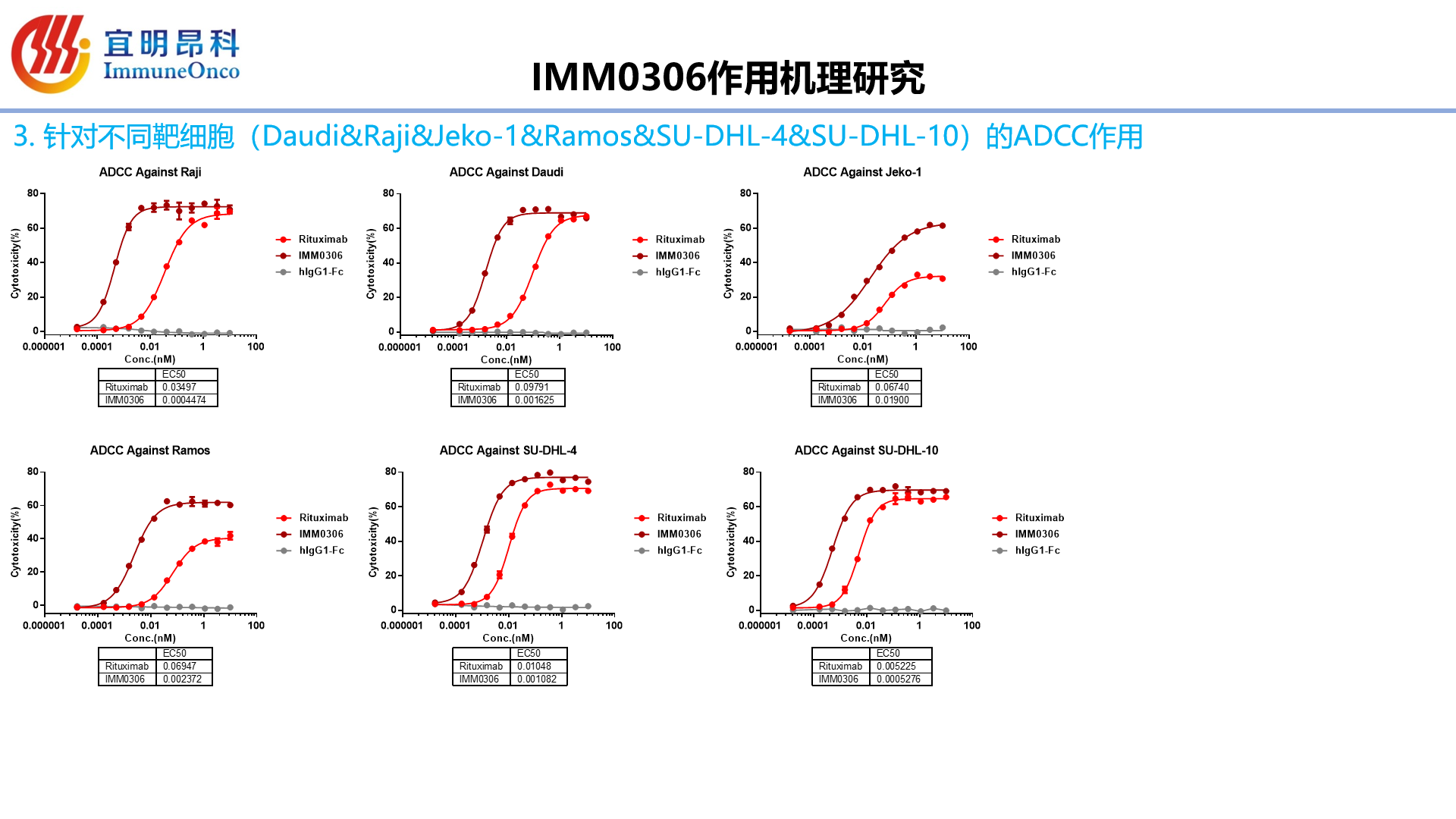


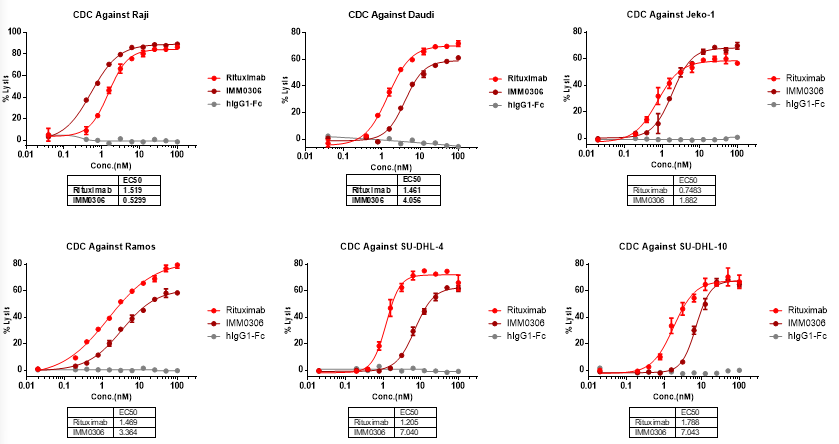
**Figure S12. IMM0306 induces lower CDC activities than rituximab on various tumor cells, including Raji, Daudi, Jeko-1, Ramos, SU-DHL-4, and SU-DHL-10.**

**Figure S13. Efficacy of IMM0306 in the xenograft of Daudi tumor mouse model.**


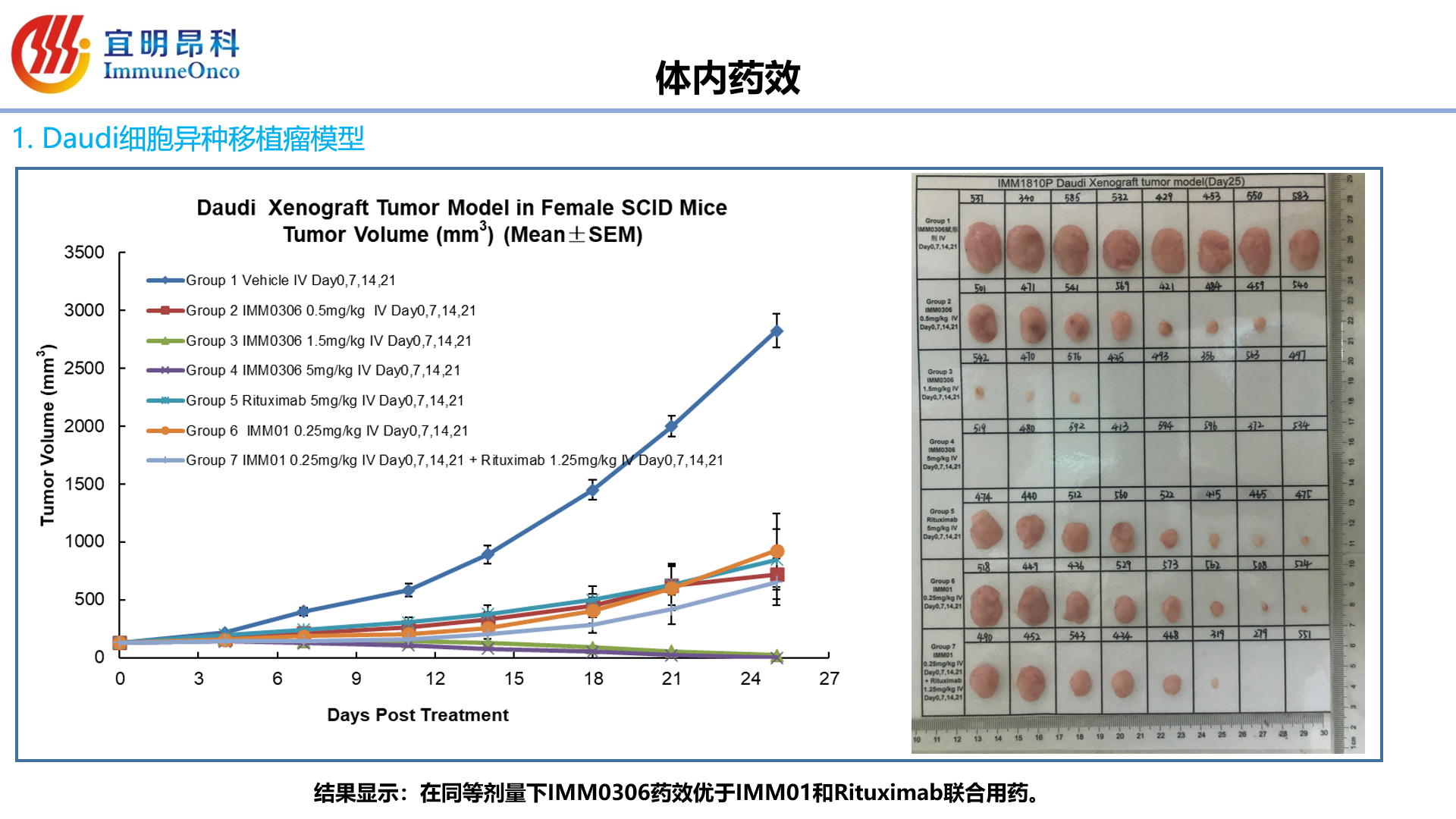
The efficacy of IMM0306 (0.5mg/kg) is better than that of IMM01 (0.25mg/kg). IMM01 is a novel SIRPα-Fc fusion protein with dual anti-tumor activities by targeting the CD47/SIRPα signal pathway via blocking the “don’t eat me” signal and activating the “eat me” signal (reference 14).

**Figure S14. Raji-Luc orthotopic transplantation model in CB17 SCID mice**

CB17-SCID mice transplanted with Raji-Luc were subjected to bioluminescent imaging. Bioluminescence for Raji-Luc engrafted mice was quantified (n=10 per study group).

**1**

**2**

**3**

**4**

**5**

**6**

**1. Vehicle**

**3. Rituximab (0.8mg/kg)**

**2. IMM0306 (1mg/kg)**

**4. Lenalidomide (3mg/kg)**

**5. IMM0306 + Lenalidomide (1 + 3mg/kg)**

**6. Rituximab + Lenalidomide (0.8 + 3mg/kg)**


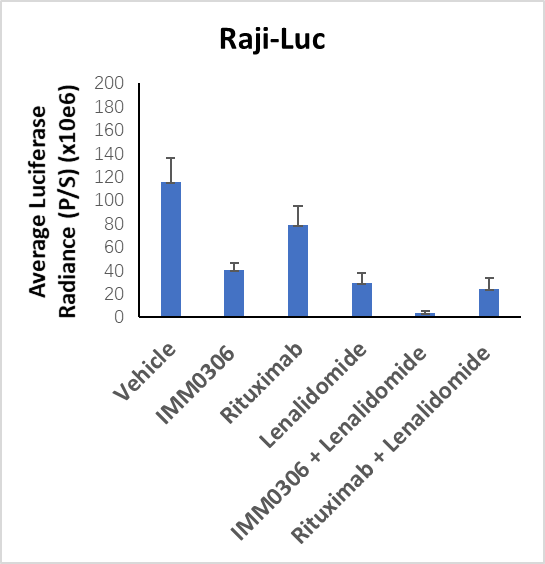


**Figure S15. Mechanism of action of IMM0306 versus action of CD47 mAb and CD20 mAb**


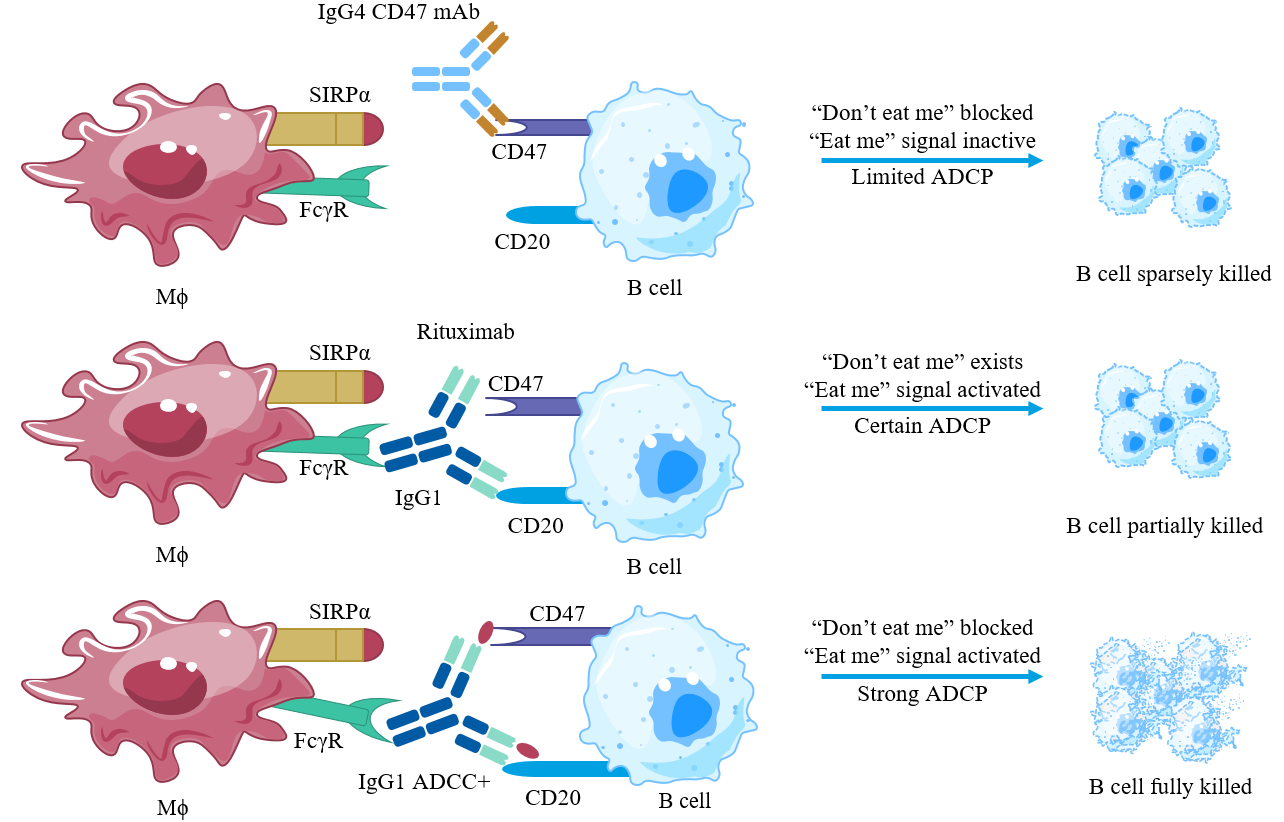

Supplement: Supplementary file 1 — Supplement file and figures S1-S15 [file 41375_2022_1805_MOESM1_ESM.docx]
